# Rheological, Morphological and Mechanical Studies of Sustainably Sourced Polymer Blends Based on Poly(Lactic Acid) and Polyamide 11

**DOI:** 10.3390/polym8030061

**Published:** 2016-02-26

**Authors:** Fatma Walha, Khalid Lamnawar, Abderrahim Maazouz, Mohamed Jaziri

**Affiliations:** 1 Laboratoire Electrochimie et Environnement, ENIS, Université de Sfax, 3038 Sfax, Tunisie; walha.fatma@gmail.com (F.W.); Mohamedjaziri2003@yahoo.fr (M.J.); 2 Université de Lyon, Institut National des Sciences Appliquées de Lyon (INSA-LYON), CNRS, F-69361 Lyon, France; 3 Ingénierie des Matériaux Polymères IMP@INSA, UMR 5223, Lyon F-69621, France; 4 Laboratoire de Mécanique des Contacts et des Structures LaMCoS, UMR 5259, Lyon F-69621, France; 5 Hassan II Academy of Science and Technology, 10 100 Rabat, Morocco

**Keywords:** biosourced polymers, compatibilization, reactive extrusion, rheology

## Abstract

The objective of this study was to gain a deep understanding of composition and compatibilization effects on the properties of entirely sustainably sourced polymer blends based on polylactide (PLA) and polyamide 11 (PA11). Generally, PLA cannot challenge regular commodity polymers due to its weak thermo-mechanical properties and its poor elongation properties. With this work, however, we present a promising route to overcome these drawbacks in order to enhance the processability of PLA: blending the polymer with various compositions of other ductile biopolymers such as PA11, as well as mixing PLA/PA11 blends with various amounts of a chain extender, Joncryl ADR^®^-4368, containing reactive epoxy functions, in a laboratory-scale twin-screw extruder. The effects on the rheological, morphological and mechanical properties were investigated. Results showed that a “self compatibilization” between PLA and PA11 chains can occur but it was found to be insufficient, contrary to recent work reported in the literature. The role of Joncryl as a compatibilizer for the PLA/PA11 system has been demonstrated by the significant decrease of particle size and interfacial tension as well as the improvement of ductile properties. Moreover, a new relaxation peak appeared in the relaxation spectrum, indicating the generation of a copolymer at the polymer-polymer interface.

## 1. Introduction

In the past decade, biopolymers or polymers issued from renewable resources have attracted significant attention for their potential to reduce dependence on petroleum-based materials and their potential applications in packaging, the medical field and the agricultural fields [1,2,3].

Among these sustainably sourced polymers, polylactide (PLA) has proven to be the most attractive and useful choice since it has a good cost structure, the best availability and excellent transparency [4]. Most industrial PLAs are linear thermoplastic polymers that are biocompatible, resorbable and biodegradable under industrial composting conditions [5,6]. It displays many good properties such as a high tensile strength and rigidity, as well as good grease and oil resistance [7].

Despite all these advantages, PLA suffers from poor thermal stability, significant brittleness and low melt strength, which limit its wide-spread use, especially for packaging applications (film blowing) [8,9]. To overcome these drawbacks, many approaches have been established, including copolymerization, compounding with nanofillers and blending with other polymers [10,11,12]. In fact, the blending of different polymers is an efficient and cost-effective strategy for developing new polymeric materials with diversified and desirable properties [13].

Many studies have focused on blending PLA with other biopolymers (chitosan, starch, PHBV, PBAT, PHB) [14,15,16,17,18,19,20]. Numerous papers have been dedicated to the improvement of mechanical properties by physical compounding and mechanical compatibilization [3,21,22]. Recently, polyamide 11, which it is a bio-sourced polymer, was used in a binary blend to improve the thermo-mechanical and barrier properties of PLA [2,23,24]. PA11 is derived from renewable resources (castor oil) and presents excellent performances such as high impact strength, high thermal stability and good chemical resistance. However, the problem of immiscibility and incompatibility between polymers can lead to inferior properties.

Stoclet *et al.* (2011) [23] studied the morphology and mechanical properties of PLA/PA11 blends. Despite their incompatibility, the authors predicated a good self-compatibilization between the two polymers highlighted by the micronic and sub-micronic dispersion of the minor phase in the major one. However, the elongation properties of the studied blends remained low. No correlation with rheological properties was carried out.

In order to enhance the mechanical properties, the incorporation of a compatibilizing agent is necessary. Rakhi Patel *et al.* (2013) [24] have studied the properties of PLA/PA11 blends in the presence of titanium isopropoxide as a catalyst to investigate potential compatibilization reactions. They also confirmed that the system remained immiscible and that the compatibilization reactions, if present, were hidden by degradation reactions. According to Wenyong Dong *et al.* (2013) [2], the use of ethylene glycidyl methacrylate-*graft*-styrene-*co*-acrylonitrile rubber (EGMA-G-AS) in PLA/PA11 blends gives a salami structure without any decrease of the domain size due to the fact that the rubber is not located at the interface. As a result, it does not take on a compatibilizing role for PLA and PA11. Moreover, this salami structure leads to better mechanical properties especially with regard to ductility and impact strength. Unfortunately, the use of EGMA-G-AS reduced the ratio of the sustainably sourced phase in the blend. Hence, the use of other kinds of compatibilizers to obtain a finely dispersed phase and to improve the cohesion between matrix and dispersed phase is necessary.

Although significant efforts have been dedicated to the study of the PLA/PA11 system, the evolution of the morphology with the composition and the rheological properties (viscosity and elasticity ratio) of each phase has yet to be understood and established, especially in the presence of a compatibilizer. Meanwhile, Rasha *et al.* (2014) [19] studied the interfacial properties of PLA/PBAT in the presence of multifunctionalized epoxy, Joncryl^®^, using linear and nonlinear rheology. The authors confirmed the role of a multifunctionalized epoxy as the compatibilizing agent.

This paper presents the effects of incorporating a multifunctional epoxide as well Joncryl^®^ on the properties of binary PLA/PA11 blends. Despite the interesting nature of the research discussed above, it was of no help when attempting to comprehend both the morphological generation and rheological properties with the incorporation of Joncryl in PLA/PA11 blends of various compositions. Through this work, we present a promising route to overcome the lower melt strength and enlarge the processability window of PLA by blending it with various compositions of other ductile biopolymers such as PA11 and with Joncryl as a chain extender of the two polymers. Two main methods of mixing were used: the first one consisted of introducing all compounds simultaneously in the extruder and the second one consisted of modifying PLA by premixing it with Joncryl and then adding the PA11.

Chain extension/branching phenomenon are expected to improve the melt strength and elongation properties for blown extrusion process. The focus to use Joncryl was also to compatibilized PLA/PA11 blends. Joncryl ADR@4368 was selected because it contains reactive Glycidyl methacrylate (GMA)/epoxide functions which can react with both hydroxyl and carboxyl reactive functional end chains of PLA and amine and carboxyl end groups of PA11.

Indeed, phase diagrams and morphological developments depending on the composition both with and without Joncryl were assessed and are discussed. Shear rheology in both linear and nonlinear regimes was developed to understand the morphological changes and the induced mechanical properties.

## 2. Experimental Section

### 2.1. Materials

**Figure 1:**
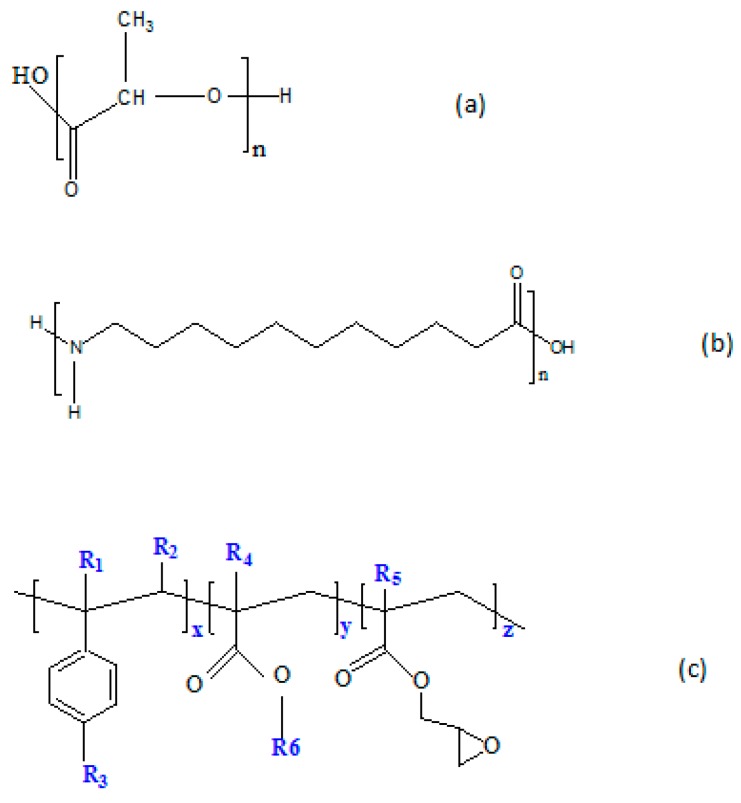
Chemical structure of (**a**) PLA, (**b**) PA11 and (**c**) Joncryl ADR^®^-4368, and the general structure of the styrene-acrylic multifunctional oligomeric chain extenders. R1–R5 are H, CH3, a higher alkyl group or combinations of them; R6 is an alkyl group. X, Y and Z are between 1 and 20.

The polylactide (PLA grade 2003D) used in this study was in the form of pellets and purchased from NatureWORKS (Minnetonka, MN, USA). It is a semi-crystalline grade with approximately 4% d-lactic acid enantiomer. The polyamide 11 (PA11 grade BESNO P40 TL) was produced by Arkema (Colombes, France) under the trade name Rilsan. A commercially modified acrylic copolymer with epoxy functions (Joncryl ADR^®^-4368) was obtained from BASF (Ludwigshafen, Germany). Its average functionality on epoxide is 9. Joncryl is accepted by the Food and Drug Administration for food packaging. Table 1 shows some properties of the materials used and Figure 1 depicts their chemical structures.

The weight-average molecular weight (*M*_w_), for both neat and modified polymers, was measured using size exclusion chromatography (SEC) consisting of a VARIAN prostar chromatograph. The latter is made of a RHEODYNE injector, two Mixed-PL gel columns (G4000 HXL to G1000 HXL) with a porosity of about 50–100,000 A°, and an RI-101 refractive index detector. The samples were initially dissolved in tetrahydrofuran (THF) at room temperature. Polystyrene having higher Mw was used to generate the calibration curve. The tests were conducted using THF with a flow rate of 0.5 mL/min. The molar masses of PA 11 were measured at 30 °C in hexafluoroisopropanol (HFIP) with a flow rate of 0.75 mL/min and calibration with PMMA standards.

### 2.2. Blends Preparation

**Figure 3:**
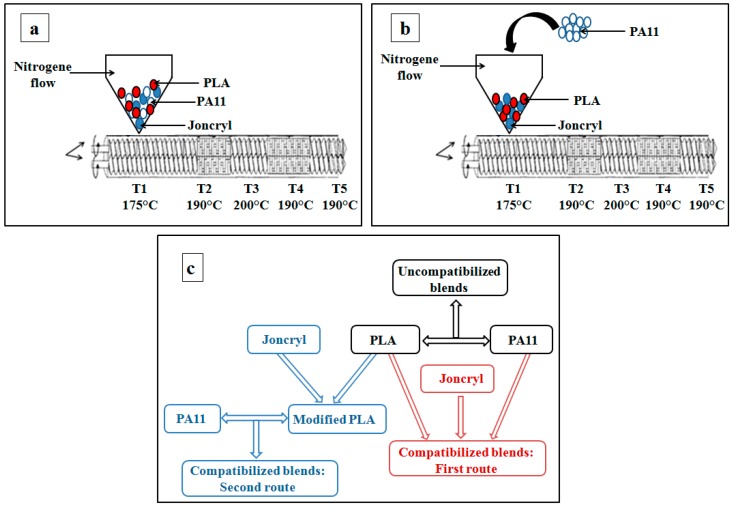
The two strategies of mixing using a corotating twin-screw extruder with a 16-mm diameter: (**a**) one-step mixing of PLA, PA11 and Joncryl; (**b**) mixing PA11 with modified PLA; (**c**) synopsis of the experiment.

Before compounding, the materials were pre-treated by drying under vacuum at 40 °C for 48 h. Then the blends were prepared in a co-rotating twin screw extruder (Thermo Electron Polylab System Rhecord RC400P, Courtaboeuf, France) with a screw diameter of 16 mm and an L/D ration of 25:1 (Figure 3). A sealed hopper was used and kept under nitrogen atmosphere to avoid oxidation and hydrolytic degradation during the blend preparation. The parameters of extrusion were: nitrogen atmosphere, screw speed = 40 rpm, residence time = 3 min, temperature profile = 175, 190, 200, 190 and 190 °C from the feed zone to the die.

Two different mixing series were used: (I) one-step mixing of all components PLA, PA11 and Joncryl; (II) premixing of PLA and Joncryl until an equilibrium state (stability of the torque) followed by addition of PA11 and mixing for 3 min. The blends were then quenched in cold water and granulated. For the first approach PLA, PA11 and Joncryl were mixed simultaneously in the twin screw extruder. The blends were prepared if necessary on two steps and they have been re-granulated to stabilize the couple. The speed rotor was adapted depending of the strategy and the total residence time was set to 3 min. For the second approach PLA and Joncryl were premixed (in the molten state) until stability of the torque (for about 2 min) and then adding in the twin screw the PA11. All the specimens were recorded after extrusion and studied by rheology according to the dynamic time sweep tests. No increase of viscosity was noted and the studied systems were assumed to reach an equilibrium state. 

After drying at 60 °C for 12 h, the blend granules were molded, using an injection press at a temperature of 190 °C and a pressure of 150 bar, into disk-shaped specimens of 2 mm thickness with a diameter of 25 mm. The compositions of the studied blends are given in Table 2.

### 2.3. Experimental Methods and Procedures

#### 2.3.1. Scanning Electron Microscopy (SEM)

Scanning electron microscopy was used to describe the morphology of the PLA/PA11 blend. The morphological properties were examined using a Hitachi S3500 microscope (Tokyo, Japan). Samples were cryogenically fractured in liquid nitrogen and coated with gold-palladium to avoid electrostatic charging for the duration of the analysis.

#### 2.3.2. Rheological Properties

- Small amplitude oscillatory shear (SAOS)

Linear viscoelastic properties were investigated on a stress-controlled rotational rheometer TA Instruments DHR-2 using a plane–plane configuration, a plate diameter of 25 mm and a gap inferior to 2 mm. Measurements were done at 190 °C under nitrogen to avoid thermal degradation of the neat polymers and their blends. The linear domain, executed at 0.1 rad/s, expanded from 0.2 to 1000 Pa. Dynamic frequency sweep tests were performed at 20 Pa over an angular frequency range of 10^−1^–628 rad/s.

- Step strain experiments of stress relaxation

Stress relaxation after single-step strain measurements were performed in the same DHR-2 stress-controlled rheometer using a parallel-disk geometry. Different strain amplitudes ranging from 5% to 50% were applied in both linear and nonlinear viscoelastic regimes.

Start-up shear experiments were carried out in the DHR-2. In this case, the strain rate was increased instantaneously at time zero and the transient shear stress was recorded over time. A shear rate of 1 s^−1^ was applied. All measurements were performed at 190 °C under a nitrogen atmosphere.

#### 2.3.3. Tensile Properties

Mechanical properties were determined using an Instron machine according to the ASTM method D638 under ambient temperature (25 °C) with a cross-head speed of 5 mm/min. The dimensions of the dumbbell specimen was 20 mm in length, 4 mm in width and a thickness of 2 mm. For each blend, five samples were examined.

## 3. Results and Discussion

### 3.1. Interfacial and Morphological Properties

The main focus of this section was to explore a PLA/PA11 blend system based on its morphological and interfacial properties. Firstly, the effect of varying the composition on the final blend properties was investigated. Secondly, we studied the impact of incorporating various amounts of a multifunctional epoxide (Joncryl) on the interfacial properties of PLA/PA11 blends using two different mixing strategies: (I) *in situ* reactive extrusion of all components at the same time; and (II) modification of PLA with epoxide functions followed by addition of PA11.

- Uncompatibilized PLA/PA11 blends

**Figure 4:**
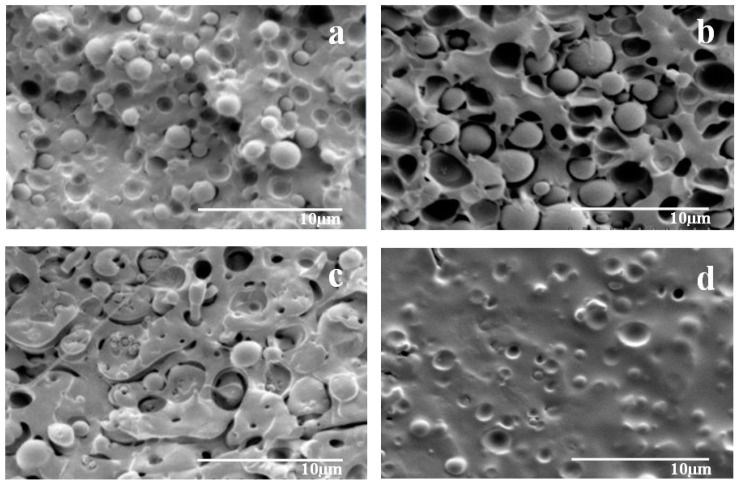
SEM micrographs of PLA/PA11 blends with various concentrations (**a**) 80/20, (**b**) 60/40, (**c**) 40/60, (**d**) 20/80.

SEM micrographs of PLA/PA11 blends at various concentrations are presented in Figure 4. It can be clearly seen that the morphological properties were affected by the compositional changes in the blend. SEM analyses illustrated a two-phase morphology with a nodular structure. At the extremes of the composition range (80/20) and (20/80), spherical particles of the minor phase were dispersed in the major one. The repartition of these particles was quite uniform and their size distribution seemed rather narrow. Most of the droplets remained in the structure, while others were pulled out during fracture leaving empty cavities.

In the case of the (60/40) blend, it can be seen that the size of the PA11 particles increased as its proportion of the composition was raised. In fact, the shape of the particles evolved toward ellipsoidal and their size distribution became larger. The morphology observed in the (40/60) blend revealed the appearance of a co-continuous structure; the presence of PLA aggregates and a coalescence of globules into larger ones. The amount of pulled-out particles was considerably lower, which was highlighted by a reduced number of cavities from the extraction of droplets upon rupture. The obtained morphology was believed to indicate the absence of miscibility between the two polymers PLA and PA11 as well as the presence of high interfacial tension in the blends. Self-compatibilization, as presumed in the literature, seemed to be limited and insufficient.

- Compatibilized PLA/PA11 blends (First approach)

In this paper, two different mixing sequences were studied. The first approach is considered one step of blending, with all components being mixed in the extruder simultaneously. The second one (Second approach) consists of modifying PLA by premixing it with Joncryl in the extruder until an equilibrium state and then adding PA11 and mixing for 3 min. Increasing melt strength and elasticity of PLA are suspected to influence compatibilisation properties. Thus, the obtained blends with the second route were called “Modified PLA/PA11 blends”.

**Figure 5:**
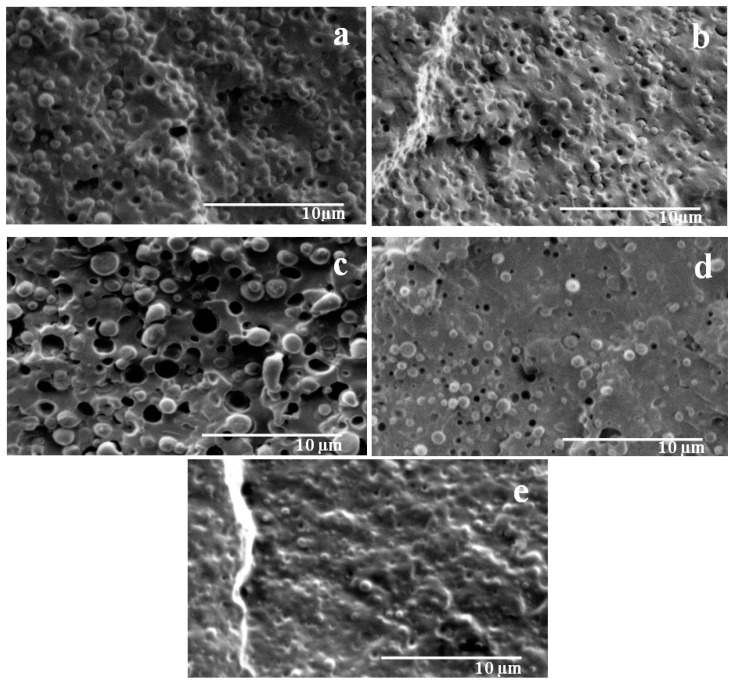
SEM micrographs of PLA/PA11/Joncryl blends (**a**) (80/20/0.7), (**b**) (80/20/1), (**c**) (60/40/0.7), (**d**) (40/60/0.7), (**e**) (20/80/0.7).

For the PLA/PA11/Joncryl blends obtained with one-step mixing, the SEM micrographs presented in Figure 5 illustrate (i) a notable reduction of the average size of dispersed particles due to a lower interfacial tension; (ii) a significant narrowing of the size distribution of the dispersed phase; (iii) a remarkable decrease of voids at the interface and of empty cavities corresponding to extracted particles; (iv) a better dispersion and more spherical particles of the minor phase; and (v) the disappearance of the co-continuous structure for the 40/60 PLA/PA11 blend.

- Modified PLA/PA11 blends (Second Approach)

**Figure 6:**
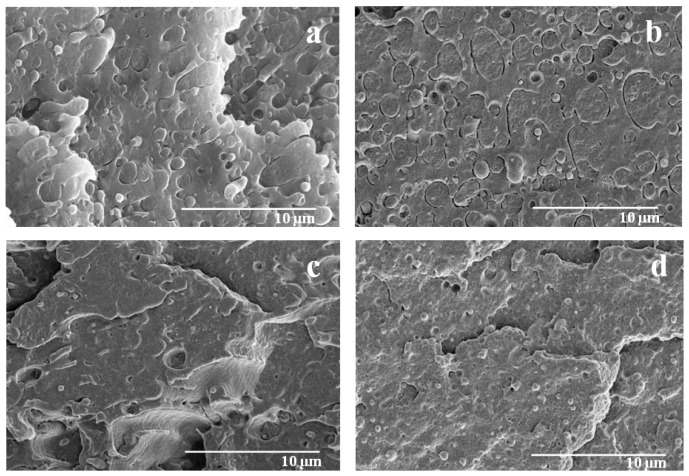
SEM micrographs of PLA_Joncryl/PA11 blends (**a**) (80_0.7/20), (**b**) (60_0.7/40), (**c**) (40_0.7/60), (**d**) (20_0.7/80).

This method consisted in modifying PLA by premixing it with 0.7% and 1% Joncryl until an equilibrium state (stability of the torque) was reached, and then adding PA11 and mixing for 3 min. The SEM micrographs in Figure 6 depict that the second mixing approach (modified_PLA/PA11 blends) led to a good adhesion between the matrix and the dispersed phases. This result is highlighted by the total disappearance of the gaps at the interface and the empty cavities corresponding to extracted particles indicating the decrease of the interfacial tension. Consequently, it was concluded that by modifying the macromolecular PLA chains, the studied blends became much more compatible.

- Effect of Joncryl on the interfacial tension properties of the PLA/PA11 blends

**Figure 7:**
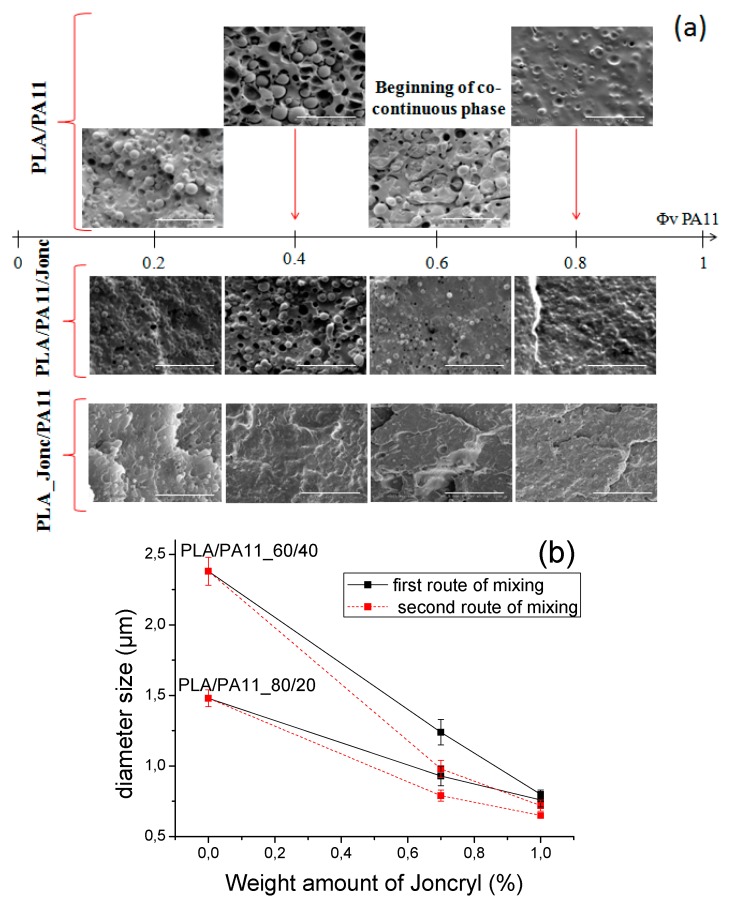
(**a**) Evolution of the morphological structure of PLA/PA11 blends with and without Joncryl as a function of the composition. (**b**) The average diameter of the dispersed particles *versus* the concentration of Joncryl.

The obtained morphology (Figure 7a) confirmed that the cohesion betwen the PLA and PA11 phases was significantly improved using the two routes of compatibilization. In fact, we note the disappearance of the co-continuous phase, empty cavities and interfacial gaps. Also, the particles seemed to be better dispersed in the matrix phase and their sizes decreased remarkably.

To confirm this decrease, the evolution of the average particle sizes was plotted as a function of the concentration of Joncryl (Figure 7b). The number-average diameter of the particles was determined by the image processing software ImageJ (NIH, USA, http://imagej.nih.gov/ij/), using Equation (1):

(1) $$D_{p}=\frac{\sum n_{i}D_{I}}{\sum n_{i}}$$

where *n*_i_ is the number of dispersed domains with a diameter Di.

It can be seen that for blends without glycidyl methacrylate (GMA) functions, the particle diameter increased for higher PA11 concentrations, passing from 1.48 µm for the 80/20 PLA/PA11 blend to 2.38 µm for the 60/40 PLA/PA11 blend. There were two major reasons for the larger particle size: (1) a large difference between the viscosity of the matrix and the dispersed phase and (2) a coalescence phenomen caused by the poor interfacial adhesion between the matrix and the nodules. With the incorporation of a multi-functionalized epoxide, we obtained a significant decrease in diameter indicating the suppression of coalescence due to the chemical reactivity of the epoxide with the polymers of the blend. This decrease was more pronounced with the second strategy of mixing indicating that blending PA11 with modified PLA was much more efficient for improving the compatibility.

The previous results revealed that Joncryl acted as a compatibilizer. In order to confirm this role, the quantification of the interfacial tension was followed using the Taylor model (Equation (2)).

(2) $$D=\frac{4\alpha (\frac{\eta_{d}}{\eta_{m}}+1)}{\overset{\cdot }{\gamma }\eta_{m}(\frac{19}{4}\times \frac{\eta_{d}}{\eta_{m}}+4)}$$

Here, D is the particle diameter, α is the interfacial tension, η_m_ is the viscosity of the matrix phase, η_d_ is the viscosity of the dispersed phase, and $\dot{\gamma }$ is the shear rate. The latter was taken here as 1 s^−1^. The equation is derived from the Taylor model. In two-phase blends in the Newtonian region, the structural evolution is a competition, during flow, between the hydrodynamic deforming stresses that tend to deform the drops and interfacial forces (α/*R*v) that resist the stress or deformation and tend to recover the initial shape of the drop. Thus, the shear rate was chosen to have a higher capillary number Ca than the critical one. This shear rate is chosen as a rule of the trust in our case, as PLA is in the Newtonian region and at the lower frequency region of PA 11 to minimize effect of shear and especially to have a viscosity ratio lower than 4, as supported by Grace’s data ( *cf.* Al-Itry *et al.* [19]).

Table 3 summarizes the obtained values of interfacial tension. It was demonstrated that the presence of GMA functions decreased the interfacial tension from 2.57 to 1.61 mN/m when using the first approach and to 1.37 mN/m with the second one.

The morphological study as well as the obtained values of interfacial forces confirmed that Joncryl acted as a compatibilizer. The challenge in the rest of the study was to understand the effect of this morphology on the rheological and mechanical properties of the obtained blends.

### 3.2. Shear Rheological Properties

#### 3.2.1. Small Amplitude Oscillatory Shear (SAOS)

- Uncompatibilized PLA/PA11 blends

**Figure 8:**
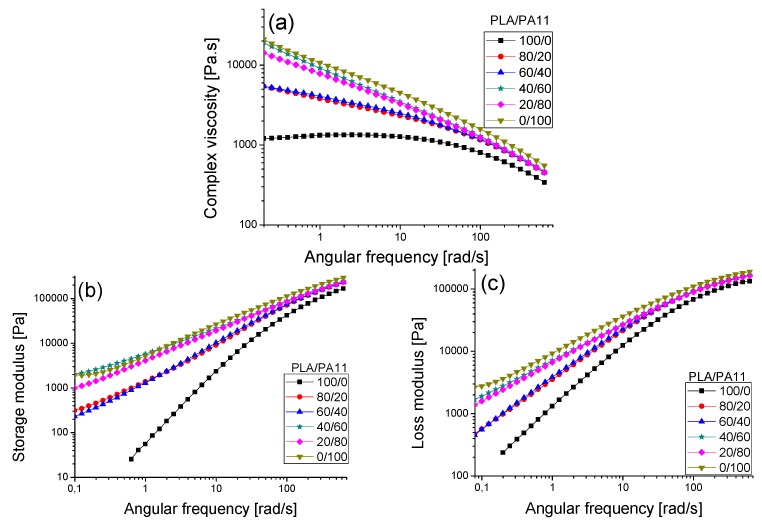
(**a**) The complex viscosity modulus *versus* the angular frequency for PLA, PA11 and PLA/PA11 blends at 190 °C at various concentrations. The storage modulus (**b**) and loss modulus (**c**) *versus* the angular frequency at 190 °C for PLA, PA11 and the PLA/PA11 blend.

Figure 8 illustrate the evolution of the complex viscosity modulus η*, the storage modulus G’ and the loss modulus G’’ *versus* angular frequency of the neat PLA and PA11 as well as their respective blends at 190 °C. Polylactide displayed a Newtonian behavior at low angular frequencies (<10 rad/s). Beyond 10 rad/s, a shear-thinning behavior was observed, highlighted by the decrease of the complex viscosity modulus. In contrast and despite its lower molecular weight than PLA, polyamide 11 showed a shear-thinning behavior in the whole angular frequency range. Its complex viscosity modulus was quite a bit higher than that of polylactide. Indeed, this observation corroborates the results of Stoclet *et al.* [23]. Therefore, the higher viscosity of PA11 could be explained by its higher polydispersity (≈2.19) compared to PLA (≈1.95). Furthermore, we must keep in mind that the intermolecular interactions as well some hydrogen bonding between the macromolecular chains in the PA11 matrix affect significantly its rheological behavior. Moreover, the “dynamic” hydrogen (H) bonding between the amides groups of the PA11 chains that exist in the melt, and the molecular weight between entanglements that is much lower in PA11 (*M*e about 2000 g/mol) than in PLA (*M*e ~ 9000 g/mol) [25,26].

The PLA/PA11 blends presented a higher shear-thinning behavior than pure PLA. Their rheological behavior seemed to be controlled by PA11 even at lower concentrations. Furthermore, the complex viscosity moduli of the studied blends were situated between the values for the neat polymers. Indeed, they displayed an intermediate rheological behavior without any negative deviation.

In addition, according to Figure 8b,c, in the whole angular frequency range considered, PA11 demonstrated a highly viscous and elastic behavior. Moduli for the PLA/PA11 blends were located between the values obtained for the neat components. It is clearly highlighted that the viscous and elastic behaviors of these blends were significantly influenced by those of PA11 even at low quantities in the blend (20% of PA11). This rheological behavior was underlined by the change of G’ and G” slopes. Moreover, a higher polyamide 11 content increased both the storage modulus G’ and the loss modulus G’’. This increase was much more pronounced with the storage modulus G’ in the field of low angular frequencies. These results confirmed the efficiency of PA11 in improving the melt viscosity and elasticity of PLA.

**Figure 9:**
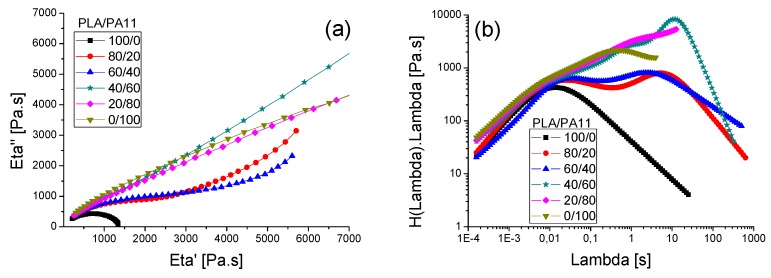
(**a**) Cole-Cole plots at 190 °C for PLA, PA11 and PLA/PA11 blends; (**b**) Relaxation spectra of PLA, PA11 and PLA/PA11 at 190 °C.

Figure 9a presents the plots of η” *versus* η’, which are typical Cole–Cole plots. A semicircular shape was obtained for neat PLA with a relaxation time at 0.012 s. However, for PA11, no arc shape was seen even at low frequencies indicating a very long relaxation time for this polymer. For the PLA/PA11 blends, we observed that the addition of PA11 led to a significant increase in relaxation time which indicates that the properties of the blends followed those of the dispersed phase. This result was in line with that for the viscoelastic behavior (variation of G’ and G” *versus* angular frequencies).

For additional information on the relaxation phenomena, the relaxation spectrum (λH(λ) *vs.* λ) is presented in Figure 9b. Both Ǵ and G” were used to calculate the stress relaxation spectrum (λH (λ)) from the dynamic moduli using the non-linear regularization method (Honerkamp and Weeze 1993) according to Equation (3):

(3) $$G*(\omega ,\lambda )=\int_{-\infty }^{+\infty }\frac{H(\lambda )i\omega \lambda }{\lambda (1+i\omega \lambda )}d\lambda$$

Here, “ω” is the angular frequency and “λ” is the relaxation time;

$$G\prime (\omega )=\int_{-\infty }^{+\infty }H(\lambda )\frac{\omega^{2}\lambda^{2}}{1+\omega^{2}\lambda^{2}}d(\ln\lambda )$$

$$G\prime \prime (\omega )=\int_{-\infty }^{+\infty }H(\lambda )\frac{\omega \lambda }{1+\omega ²\lambda ²}d(\ln\lambda )$$

$$\eta_{0}=\int_{-\infty }^{+\infty }\lambda H(\lambda )d(\ln\lambda )$$

It was found that the relaxation of macromolecular chains of both PLA and PA11 occurred at respectively 0.012 s and 0.5 s. For the PLA/PA11 blends, we note the presence of two relaxation peaks. The first one, at shorter times (0.01 s), was related to the relaxation of the PLA matrix whereas the second one, situated at τ_s_ ≈ 2.5 s, could be attributed to the shape relaxation. The relaxation peak of PA11 was hidden for PLA-rich blends (80/20 and 60/40) and appeared with higher PA11 concentrations (60% and 80%).

Since there is no Newtonian plateau for PA11 and the modified PLA blends, the reference dynamic viscosity modulus η* (0.1 rad/s) of the neat polymers and their blends was determined as the reference value for the additivity rule (Equation (4)):

(4) $$\ln(\eta_{apparent\_blend})=\phi_{1}\ln\eta_{\left(1,0.1rad/s\right)}+\phi_{2}\ln\eta_{\left(2,0.1rad/s\right)}$$

where Φ is the volume fraction of the components (PLA and PA11) and –η_(0.1 rad/s)_ is the reference dynamic viscosity of PLA and PA11 (indexed 1 and 2 respectively).

**Figure 10:**
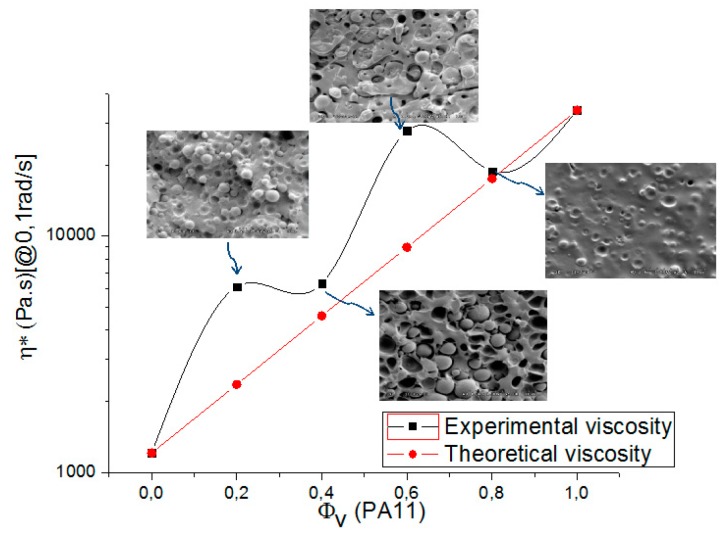
The experimental and the apparent viscosity from the additivity rule of the PLA/PA11 blends as a function of the blend composition at 190 °C.

Figure 10 illustrates the variation of these values as a function of the blend composition and theoretical viscosities obtained using the additivity rule for compatible systems.

The results show a positive deviation behavior of η_0_ from the log additivity rule for blends containing less than 80% of PA11 suggesting some phase interaction and partial self-compatibility between the polymers. For the 20/80 PLA/PA11 blend, the experimental viscosity value coincided with the theoretical one.

**Figure 11:**
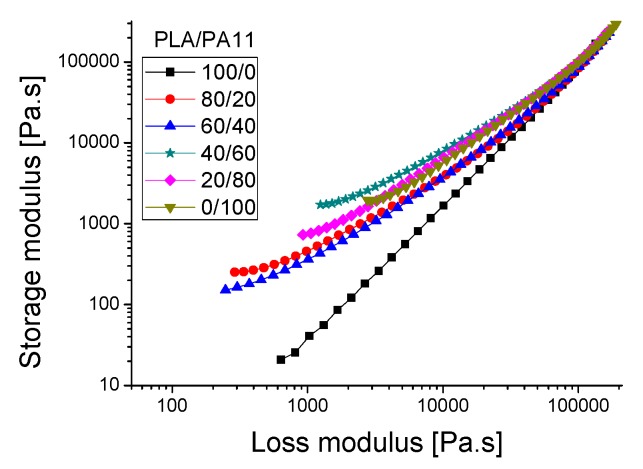
Han plot: the storage modulus (G’) *versus* loss modulus (G’’) for the PLA/PA11 blends.

The compatibility of a polymer blend in the melt state can also be determined through a Han plot, which shows a linear correlation in the plot of log G’ *versus* log G’’. In fact, the same slope is for compatible systems observed at various compositions of the pure components. Otherwise, the blend is considered to be immiscible or phase-separated [27,28]. Figure 11 displays a plot of the neat polymers (PLA and PA11) and their blends. It is clearly shown that curves of these blends exhibited a nonlinear correlation and an upturning shape at low modulus (low frequencies) which indicates nonmiscibility and incompatibility between these polymers. This result contradicts the good self-compatibility of PLA/PA11 blends proved by Stoclet G. *et al.* (2011) [23] and underlines the necessity of using a compatibilizing agent.

**Figure 12:**
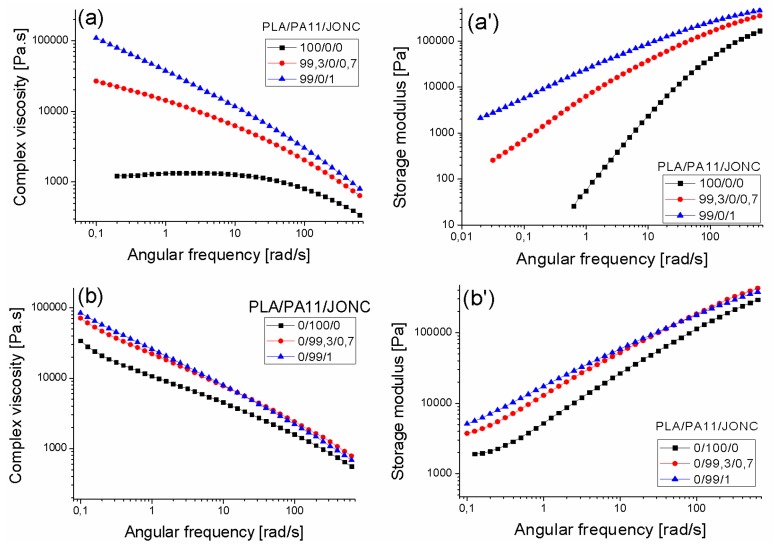
The angular frequency dependence of the complex viscosity modulus and the storage modulus at 190 °C for neat PLA (**a**,**a’**) and PA11 (**b**,**b’**) and their modified counterparts with 0.7% and 1% of Joncryl.

- *In situ* reactive extrusion of PLA/PA11/Joncryl blends (compatibilization, first approach):

Figure 12 shows that the dynamic viscosity modulus values η* increased over the whole frequency range when increasing the amount of Joncryl, which can be explained by chain extension and/or a branching phenomenon. Indeed, it can be clearly seen that the incorporation of the multi-functionalized epoxide had a noticeable effect on the rheological behavior of both neat polymers PLA and PA11. The longer and heavier chains with short chain branches created more entanglements thus resulting in higher molecular weights giving rise to higher viscosities [17,19,29,30]. Also, the chain extension with respectively 0.7% and 1% of Joncryl displayed a more pronounced shear thinning tendency of PLA and consequently shifted the Newtonian plateau to lower angular frequencies [17,31]. The same trend was observed for PA11 with 0.7% and 1% of Joncryl. Moreover, we noted a significant increase in storage modulus G’ of both PLA and PA11 with an increased content of multifunctionalized epoxide especially at low angular frequencies (Figure 12a’,b’). This highlighted the improvement of the elastic behavior of PLA and PA11. Moreover, it was found that the storage modulus for PA11 became less sensitive with an increasing Joncryl content. Furthermore, the analysis of the obtained PLA/PA11/joncryl samples upon rheology confirms also the total solubility of this system.

**Figure 13:**
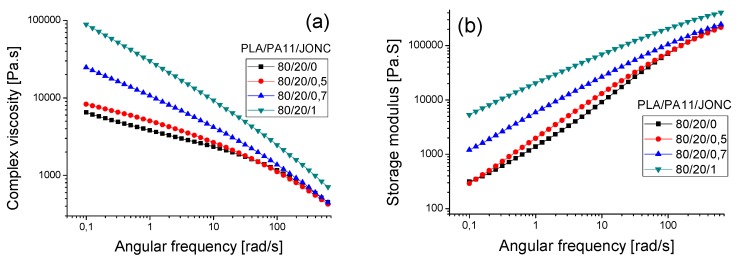

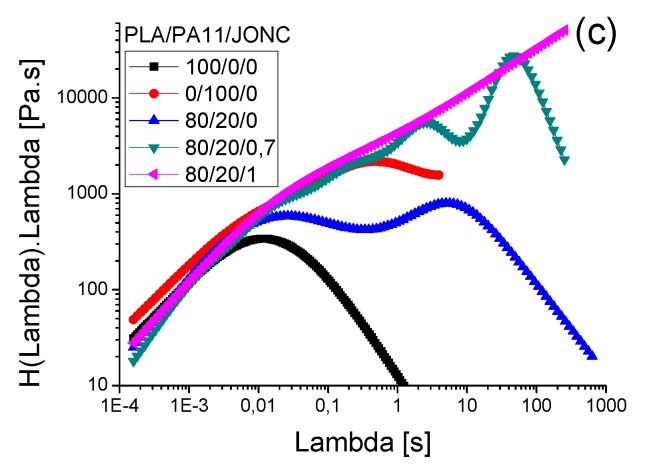
The angular frequency dependence of the complex viscosity modulus (**a**) and the storage modulus (**b**) at 190 °C for the 80/20 PLA/PA11 blend with various amounts of Joncryl, *i.e.*, 0.5%, 0.7% and 1% (**c**) relaxation spectra of neat PLA and PA11 and their uncompatibilized and compatibilized 80/20 blends with 0.7% and 1% of Joncryl.

On the one hand, Figure 13a,b illustrates a significant improvement in melt strength for the 80/20_PLA/PA11 blend with various amounts of Joncryl, suggesting that some chemical reactions took place for the neat PLA and PA11. The shear thinning behavior of PLA/PA11 blends became more pronounced with the increase in multi-functionalized epoxide content which may indicate an improvement of the melt stability during processing [32]. This phenomenon could be explained by the reaction of epoxy functions with both NH_2_ and acid chain end of PA11 and PLA, respectively [27,33,34].

In addition, an increase of the complex viscosity modulus η* with increasing Joncryl content in the blend was highlighted in the whole frequency range, indicating that the interfacial reaction increased the intermolecular interactions of the blend system (Zhang N. *et al.* 2009) [32]. On the other hand, a drastic raise in storage modulus G’ was emphasized with the chain extender concentrations. This increase, mainly at low angular frequencies, indicates for both the neat PLA and PA11, the appearance of some chain-extended/branched chains as well as an increase of the entanglement density. It should be noted that this improvement of rheological properties of the PLA/PA11 blend was more pronounced with 0.7 wt% and 1 wt % of Joncryl. However, with 0.5 wt % there was no significant change in the behavior of the PLA-PA11 blend, which may be due to insufficient compatibilization between the two polymers.

Furthermore, the relaxation spectrum of the PLA_80/PA11_20 blend compatibilized with 0.7 wt % of Joncryl (Figure 13c) clearly showed three relaxation peaks: the first one was related to the relaxation of the PLA matrix (at 0.012 s), the second peak was attributed to the shape relaxation of the dispersed PA11 phase (at 3 s) and finally an additional longer characteristic relaxation peak was visible at 50s and could be attributed to the PLA-Joncryl-PA11 copolymer relaxation. For the blend compatibilized with 1 wt % of Joncryl, the relaxation peaks disappeared, probably indicating the very long relaxation time of this compatibilized blend.

**Figure 14:**
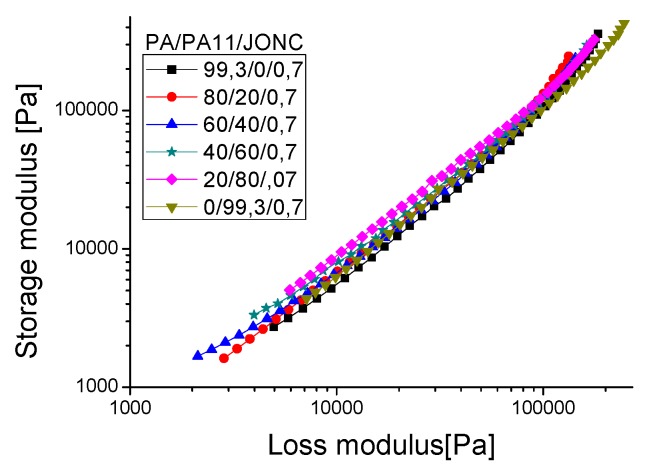
Han plot: the storage modulus (G’) *versus* loss modulus (G’’) for the PLA/PA11/Joncryl blends.

In order to ensure that Joncryl acted as a compatibilizer for the PLA/PA11 blends, the Han plot was investigated (Figure 14). Indeed, with incorporation of this multifunctionalized epoxide, the curves of the blends displayed a linear correlation and a close slope, indicating compatibility in comparison with the results of Figure 11 for PLA/PA11 blends. The results corroborate those of SEM shown before. 

**Figure 15:**
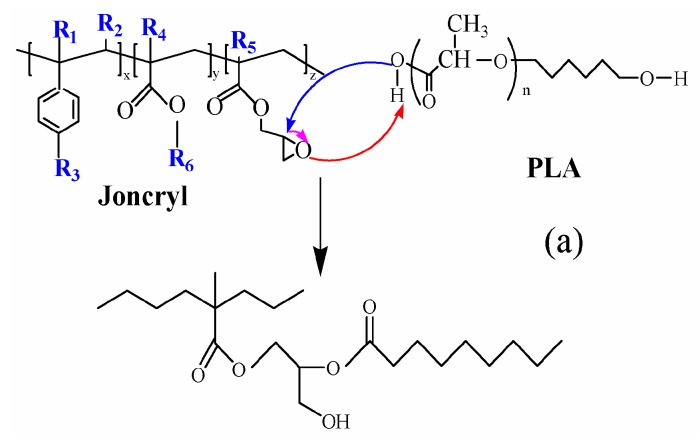

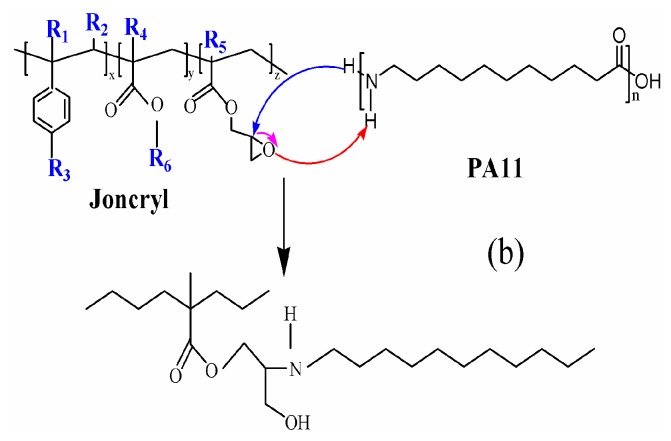
(**a**) Predicted reaction between carboxylic end group of PLA and Joncryl and (**b**) between amine end group of PA11 and Joncryl.

This result revealed once again the occurrence of chemical reactions between the glycidyl methacrylate groups of Joncryl and both the carboxyl (–COOH)/hydroxyl (–OH) end groups of PLA and carboxyl (–COOH)/amino end groups (–NH_2_) of PA11. In the case of PLA/Joncryl reaction, glycidyl esterification of carboxylic acid end groups precedes hydroxyl end group etherification [17]. Figure 15a,b respectively illustrate the predicted reactions between PLA and PA11 with Joncryl, and Table 4 lists the different reaction rates of the epoxide with other groups [26,35].

The reaction mechanisms between PLA/PA11/GMA were supported by the literature and confirmed by FTIR in our previous work [17,36]. For the clarity purpose of the present work, FTIR results were not presented here. Also, the interactions between both PLA and other polyesters as well PBAT with Joncryl have been widely discussed in our previous work (Al-Itry *et al.* [17,30]). Several possible mechanisms have been suggested. To avoid repeating results already published, we decided to not develop in detail this part in the present manuscript. To more information, the readers could refer to our previous papers. Lamnawar *et al.* [33,34,36] studied the interfacial reaction between COOH/GMA and NH2/COOH end groups with rheology coupled to FTIR or NMR. Japon *et al.* [37] allow differentiating between the reaction of carboxylic acid and hydroxyls groups with epoxyde, since epoxide groups are known to react differently with –COOH and –OH groups. In addition, the rate of reaction is smaller for the couple (Epoxy–OH) compared to the couple (Epoxy-COOH) (Lamnawar *et al.* 2008) [33]. This means that, in our case, the epoxy/GMA functions react mainly with COOH end groups of PLA, NH_2_, COOH end groups of PA11 (initially present in the end of the macromolecular chains or formed via thermal degradation mechanisms). Thanks to the coupling between rheology and spectroscopic tools, the results of Lamnawar *et al.* 2010 [36] highlighted that the main reaction mechanism was governed by the interfacial reaction between the GMA and carboxylic acid units, and not by that between GMA and amine end functions. These results are, in fact, in agreement with the findings of Orr *et al.* [38] derived from studies of the varying kinetics of the reaction between several pairs of functional groups of polystyrene.

- Reactive extrusion of modified PLA/PA11 blends: (PLA_J)/PA11 (second approach)

**Figure 16:**
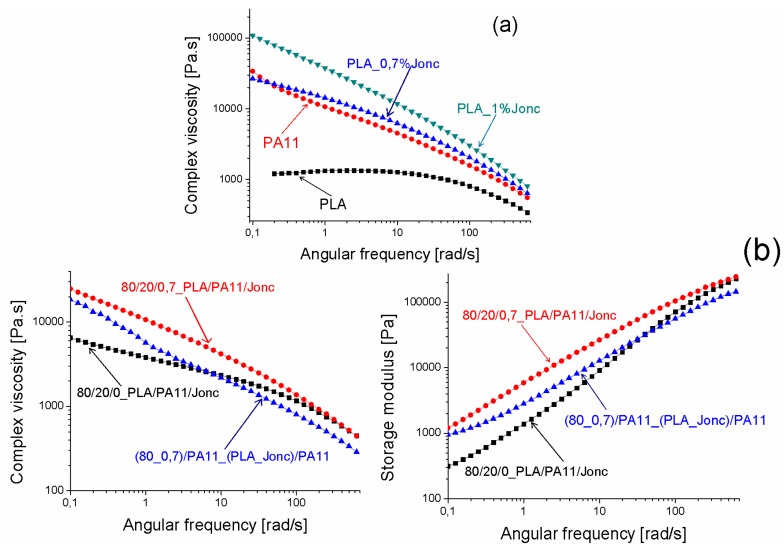
(**a**) The complex viscosity and the storage modulus *versus* the angular frequency at 190 °C of the components from the second route of mixing (modified_PLA and PA11). (**b**) The complex viscosity modulus and storage modulus *versus* the angular frequency at 190 °C for 80/20/0, 80/20/0.7 PLA/PA11/Joncryl and (80_0.7)/20 (PLA_Jonc)/PA11.

Since PA11 is a highly viscous and elastic polymer to be dispersed in PLA, the first approach described above aimed to increase the viscosity and elasticity of PLA to tailor its viscoelastic properties in order for them to be close to those of PA11, as shown in Figure 16a. Furthermore, Figure 16b illustrates the complex viscosity modulus and the storage modulus *versus* the angular frequency at 190 °C for 80/20/0 and 80/20/0.7 and (80_0.7)/20 PLA/PA11/Joncryl blends. It is clearly shown that the complex viscosity modulus and the storage modulus of the modified_PLA/PA11 blend decreased compared to that of the PLA/PA11/Joncryl blend. This was expected as a result of the epoxide functions of Joncryl in this case reacting with the carboxyl and hydroxyl groups of PLA instead of with the carboxyl and amine groups of PA11. We could also note that η* and G’ of the modified_PLA/PA11 blend were higher than those of its uncompatibilized counterpart at a low angular frequency.

**Figure 17:**
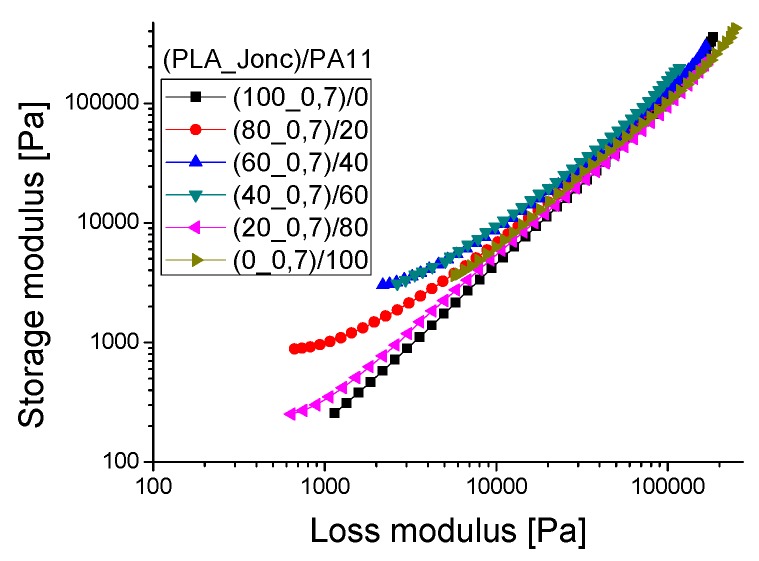
Han plot: the storage modulus (G’) *versus* loss modulus (G’’) for (PLA_Jonc)/PA11 blends at 190 °C.

A Han plot of the modified PLA/PA11 blends (Figure 17) displayed the presence of two different areas: the first one was situated at high frequency (>4 rad/s) where all blends had a single linear correlation, and the second was located at low frequency (<4 rad/s) in which all curves of these blends exhibited a nonlinear correlation, indicating nonmiscibility at low frequency.

#### 3.2.2. Step Strain and Start up Shear Experiments

**Figure 18:**
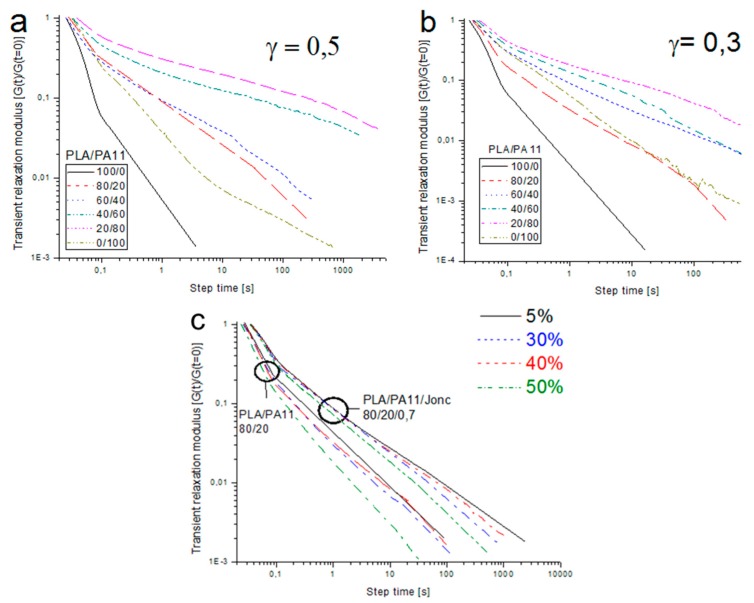
Relaxation moduli G(t,γ) of pure PLA, PA11 and PLA/PA11 blends during the application of a strain of (**a**) 5% and (**b**) 30%. (**c**) Relaxation moduli G(t,γ) of uncompatibilized (PLA/PA11_80/20) and compatibilized (PLA/PA11/Jonc_80/20/0.7) blends during application of strains at different level percentages.

Shear stress relaxation measurements G(t,γ) of the neat PLA, PA11 and their blends are presented in Figure 18a,b. The relaxation modulus was obtained at a temperature of 190 °C and two different strains: 0.05 (linear regime) and 0.3 (nonlinear regime). It can be seen that the relaxation curves of all blends contained two relaxation steps: the first faster step which probably corresponded to the relaxation of the PLA phase and the second slower step which could be related to the relaxation of PA11 and/or their interfaces.

It can be seen that the stress relaxation modulus was influenced by the blend composition and the amplitude of the applied strain. In fact, we note that G(t,γ) increased when increasing the dispersed phase concentration and when increasing the amplitude of the strain. This was probably due to larger deformations of the droplets.

Figure 18c depicts the stress relaxation moduli of the uncompatibilized and compatibilized PLA/PA11_80/20 blends. Firstly, for the shortest times, the G(t,γ) displayed a first step that could be correlated to the PLA matrix (the inelastic polymer). Secondly, it can be noted that the following relaxation steps increased and grew for long times for blends containing Joncryl, which indicated a better cohesion at the interfaces. This improved adhesion was due to the suppression of droplet coalescence and the elimination of interfacial slip in the presence of a compatibilizer. These results corroborated those of the SAOS relaxation experiments in which we demonstrated the presence of a PLA-Jonc-PA11 copolymer.

#### 3.2.3. Start-up Shear Experiments

**Figure 19:**
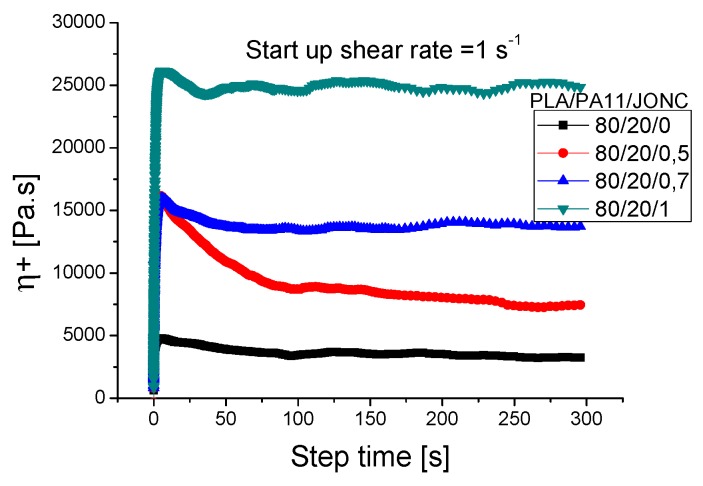
Viscosity growth (η+) upon start-up of shear for uncompatibilized and compatibilized PLA/PA11_80/20 at 1 s^−1^.

The viscosity growth function η+(γ,t) of the PLA/PA11/Joncryl blends in Figure 19 reveals the nonlinear viscoelastic behavior of the blends. The stress response increased from zero to a steady-state value. Also, a pronounced maximum (overshoot) was observed at short times. It is clearly shown that Joncryl had an important effect on the start-up experiments. In fact, at the same shear rate (1 s^−1^), the growth viscosity of the uncompatibilized blends was lower than for their compatibilized counterpart and increased with increasing Joncryl concentration. This was believed to be due to the presence of a thick droplet-matrix interface, or a droplet-matrix interphase, with the incorporation of Joncryl [19]. It is clear that the positions of the overshoots were independent of the weight fraction of compatibilizer [39], but their values depended significantly on the amount of Joncryl incorporated. This overshoot probably originated from the formed interface in the PLA-Joncryl-PA11 copolymer and the structural modification of PLA_Joncryl and PA11_Joncryl.

**Figure 20:**
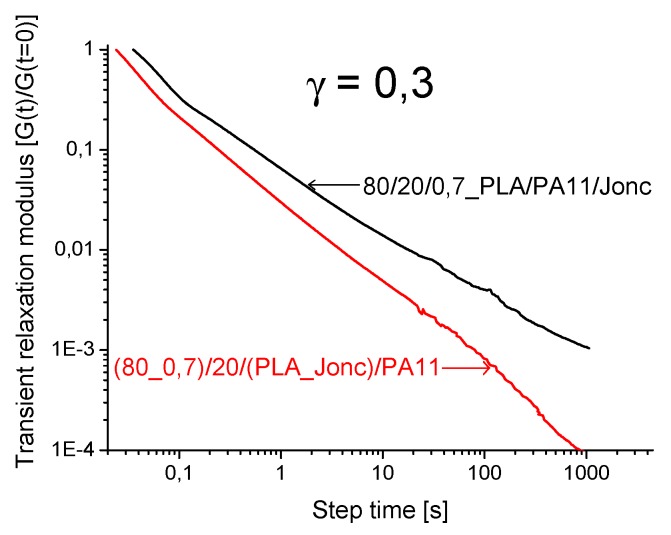
Relaxation moduli G(t,γ) of PLA/PA11/Joncryl (80/20/0.7) and PLA_Joncryl/PA11(80_0.7/20).

An example of the shear relaxation comparison of the two routes is illustrated in Figure 20. It is clearly highlighted that the relaxation modulus G(t,γ) of the PLA/PA11/Joncryl (80/20/0.7) blend was quite a bit higher than G(t,γ) of the modified PLA/PA11 (80_0.7/20) blend. This result was probably due to the modification of the structure of PLA_Joncryl and PA11_Joncryl obtained through the first approach, whereas in the second route only the PLA-Joncryl-PA11 copolymer was formed.

### 3.3. Mechanical Properties of the PLA/PA11 Blends

**Figure 21:**
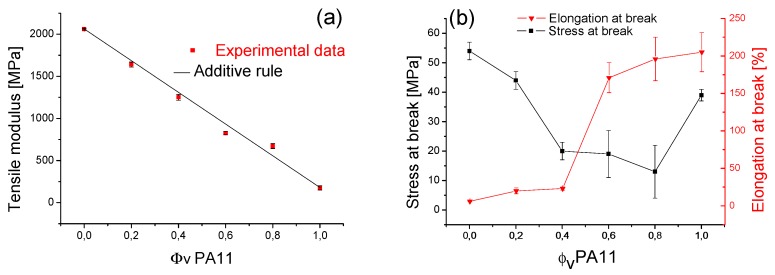
Mechanical properties (tensile modulus, stress at break and strain at break) of PLA/PA11 blends at various concentrations.

The evolution of tensile properties (tensile modulus, stress at break and strain at break) as a function of the blend composition is shown in Figure 21. It can be clearly observed that neat PLA had a high stiffness and brittleness (E ≈ 2060 MPa and ε_r_ ≈ 6%). The mechanical properties of the obtained PLA/PA11 blend changed drastically when PA11 (E ≈ 175 MPa and ε_r_ ≈ 205%) was incorporated in the mixture.

In fact, as illustrated in Figure 21a, the tensile modulus of PLA/PA11 blends covertly obeyed the simple additive mixture law (Equation (5)) and was found to be between the limiting values of the neat polymers (2060 MPa for PLA and 175 MPa for PA11).

(5) $$E_{blend}=\phi_{PLA}E_{PLA}+\phi_{PA11}E_{PA11}$$

Here, *E* is the tensile modulus and φ is the volume fraction of components.

Figure 21b shows that the brittle properties of PLA were reduced by the addition of PA11. This was an interesting find, confirming the choice of this material to improve the mechanical properties of PLA. Hence, the elongation at break increased by adding PA11, especially when it was the majority phase (e.g., at 60% of PA11, ε_r_ increased from 6% for pure PLA to 170% for the PLA/PA11 blends).

With the incorporation of Joncryl into PLA/PA11 blends (Table 5), a significant improvement of mechanical properties was obtained, especially the elongation at break which increased from 20% for the PLA/PA11 (80/20) blend to 260% for the PLA/PA11/Joncryl (80/20/0.7) blend and up to 355% for the PLA_Joncryl/PA11 (80_0.7/20) blend. This result indicates that reactivity control took place at the interface between PLA, PA11 and GMA functions and also confirms the positive effect of the multifunctional epoxide chain extender on the compatibilization between PLA and PA11. The obtained mechanical properties corroborated the results from SEM microscopy and rheological investigations.

## 4. Conclusions

Throughout this work, we have demonstrated that PLA and PA11 are immiscible and incompatible polymers, as judged from the two-phase morphology obtained and the nonlinear correlation seen in the Han plot. To improve the homogeneity of the system, we used two compatibilization methods. The first one involved reactive extrusion of PLA and PA11 with incorporation of a multifunctionalized epoxy. It was revealed, from rheological morphological and mechanical properties, that Joncryl acted as a compatibilizer. In fact, a much better adhesion between the polymers was evident from SEM micrographs with a refining morphology indicating the decrease of the interfacial tension. Moreover, a linear correlation and a close slope were seen in the Han plot, demonstrating compatibility between the phases, and a new relaxation peak at high relaxation times was detected in the relaxation spectrum corresponding to the formation of a PLA-Joncryl-PA11 copolymer. Finally, an enhancement of the ductility of PLA was highlighted by a significant increase of the elongation at break of the system.

The second route consisted in modifying PLA by adding a chain branching agent in order to increase both the viscosity and the elasticity of PLA to tailor its viscoelastic properties in order for them to be close to the behavior of PA11. The morphological structures demonstrated good adhesion between the two phases, indicating that GMA functions had reacted with PLA and that the modified system had reacted well with PA11.

## Figures and Tables

**Figure 2:**
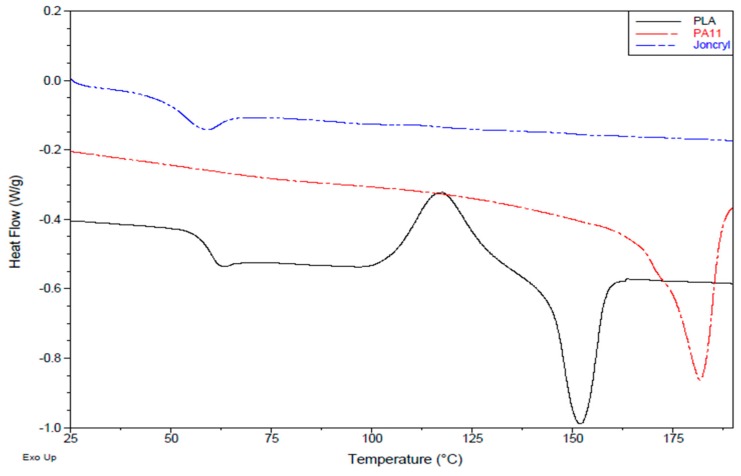
DSC thermograms (second heating cycle) of PLA, PA11 and Joncryl.

**Table 1:** Characteristics of the used materials.

Material	Density [g/cm^3^]	Melt temperature ^©^ [°C]	Glass temperature ^©^ [°C]	Average molecular weight *M*_w_ [g/mol]
PLA	1.24	155	55	202,980
PA11	1.05	180	45	25,000
Joncryl	1.08 *	-	54	6,800

**^©^** The glass and melt temperature are determined from differential scanning calorimetry (DSC) analysis at 10 °C/min (Figure 2);* This is a specific gravity as given by BASF technical data sheet.

**Table 2:** The composition and the designation of the studied blends.

Material	Blend designation	PLA [wt %]	PA11 [wt %]	Joncryl [wt %]
PLA/PA11	(100/0/0)	100	0	0
	(80/20/0)	80	20	0
	(60/40/0)	60	40	0
	(40/60/0)	40	60	0
	(20/80/0)	20	80	0
	(0/100/0)	0	100	0
PLA/PA11/Jonc Route 1	(99.3/0/0.7)	99.3	0	0.7
	(99/0/1)	99	0	1
	(80/20/0.5)	79.6	19.9	0.5
	(80/20/0.7)	79.44	19.86	0.7
	(80/20/1)	79.2	19.8	1
	(60/40/0.7)	59.58	39.72	0.7
	(40/60/0.7)	39.72	59.58	0.7
	(20/80/0.7)	19.86	79.44	0.7
	(0/99.3/0.7)	0	99.3	0.7
	(0/99/1)	0	99	1
PLA_Jonc/PA11 Route 2	(80_0.7/20)	79.3	20	0.7
	(60_0.7/40)	59.3	40	0.7
	(40_0.7/60)	39.3	60	0.7
	(20_0.7/80)	19.3	80	0.7

**Table 3:** Interfacial tension values of the compatibilized and uncompatibilized blends.

Samples	Interfacial tension [mN/m]
PLA/PA11/Jonc_80/20/0	2.57
PLA/PA11/Jonc_80/20/0.7	1.61
PLA_Jonc/PA11_80_0.7/20	1.37

**Table 4:** Reaction rate of epoxide with other groups.

Reactive couple	Reaction rate [s^−1^]
Epoxide/Amine (R–NH_2_)	260
Epoxide/Carboxylic acid (R–COOH)	18
Epoxide/Primary Hydroxyl (R–OH)	1.2
Epoxide/Secondary Hydroxyl (R–OH)	1

**Table 5:** Effect of Joncryl on mechanical properties.

Samples name	Compositions	Tensile modulus (MPa)	Elongation at break (%)
PLA/PA11	100/0	2060 ± 25	6.0 ± 0.5
	0/100	175 ± 10	205 ± 26
	80/20	1640 ± 30	20 ± 4
PLA/PA11/Jonc First route	99.3/0/0.7	2079 ± 36	5.8 ± 1
	0/99.3/0.7	192 ± 5	178 ± 13
	80/20/0.7	1662 ± 10	260 ± 15
(PLA_Jonc)/PA11 Second route	80_0.7/20	1328 ± 15	355 ± 20
